# Ventilation heterogeneity is increased in patients with chronic heart failure

**DOI:** 10.14814/phy2.12590

**Published:** 2015-10-22

**Authors:** Kirk Kee, Christopher Stuart-Andrews, Kris Nilsen, Jeremy P Wrobel, Bruce R Thompson, Matthew T Naughton

**Affiliations:** 1 Department of Medicine, Central Clinical School, Monash UniversityMelbourne, Australia; 2 Department of Allergy, Immunology and Respiratory MedicineMelbourne, Australia

**Keywords:** heart failure, multiple-breath nitrogen washout, ventilation heterogeneity

## Abstract

In the healthy lung, ventilation is distributed heterogeneously due to factors such as anatomical asymmetry and gravity. This ventilation heterogeneity increases pathologically in conditions such as asthma, chronic obstructive lung disease, and cystic fibrosis. In chronic heart failure, lung biopsy demonstrates evidence of peripheral lung fibrosis and small airways narrowing and distortion. We hypothesized that this would lead to increased ventilation heterogeneity. Furthermore, we proposed that rostral fluid shifts when seated patients lie supine would further increase ventilation heterogeneity. We recruited 30 ambulatory chronic heart failure patients (57 ± 10 years, 83% male, left ventricular ejection fraction 31 ± 12%) as well as 10 healthy controls (51 ± 13 years, 90% male). Heart failure patients were clinically euvolemic. Subjects underwent measurement of ventilation heterogeneity using the multiple-breath nitrogen washout technique in the seated position, followed by repeat measurements after 5 and 45 min in the supine position. Ventilation heterogeneity was calculated using the lung clearance index (LCI), Sacin and Scond which represent overall, acinar, and small conducting airway function, respectively. Lung clearance index (9.6 ± 1.2 vs. 8.6 ± 1.4 lung turnovers, *P* = 0.034) and Scond (0.029 ± 0.014 vs. 0.006 ± 0.016/L, *P* = 0.007) were higher in the heart failure patients. There was no difference in Sacin (0.197 ± 0.171 vs. 0.125 ± 0.081/L, *P* = 0.214). Measures of ventilation heterogeneity did not change in the supine position. This study confirms the presence of peripheral airway pathology in patients with chronic heart failure. This leads to subtle but detectable functional abnormalities which do not change after 45 min in the supine position.

## Introduction

Patients with chronic heart failure (CHF) are exposed to increased pulmonary capillary pressures which can result in injury to the pulmonary capillaries in a processed termed pulmonary capillary fracture (Tsukimoto et al. 1991). Recurrent episodes of pulmonary capillary fracture lead to changes in the lung parenchyma and peripheral vessels.

Postmortem and biopsy investigation of lung tissue in patients with CHF as well as animal models have demonstrated significant pulmonary histopathology (Kee and Naughton 2010). There is evidence of pulmonary capillary congestion with capillary dilatation (Parker and Weiss 1936) with increased capillary basement membrane thickness (Parker and Weiss 1936; Kay and Edwards 1973; Lee 1979; Haworth et al. 1988). The pulmonary vessels are thickened due to intimal thickening of the arteries and veins (Parker and Weiss 1936; Aber and Campbell 1965; Jordan et al. 1966; Huang et al. 2001) with muscularization of the arterioles and venules (Aber and Campbell 1965; Jordan et al. 1966; Haworth et al. 1988). There is accompanying circumferential fibrosis of both veins and arteries (Haworth et al. 1988). Alveolar wall changes include increased interstitial tissue (Kay and Edwards 1973), interstitial pericapillary edema (Parker and Weiss 1936; Kay and Edwards 1973), hemosiderin deposition (Jordan et al. 1966; Kay and Edwards 1973), and alveolar wall thickening due to excess collagen (Huang et al. 2001), cuboidal (as opposed to flat) epithelium (Parker and Weiss 1936; Kay and Edwards 1973), and increased type II pneumocytes (Kay and Edwards 1973; Lee 1979). Accordingly, studies have demonstrated significant pulmonary parenchymal fibrosis in CHF as well as unorganized fibrin and fibroblasts in more acute cases (Heard et al. 1968; Jasmin et al. 2003). These changes lead to compression of the peripheral airways (Haworth et al. 1988). Bronchial smooth muscle hypertrophy is also evident (Haworth et al. 1988).

Transfer factor for the lung for carbon monoxide (TLCO) falls with increasing pulmonary artery wall thickness in heart failure patients (Aber and Campbell 1965), however the effects of the parenchymal changes to ventilation heterogeneity have not been measured. As these changes occur mostly in the peripheral airways, significant changes could occur before being detected by spirometry. In fact spirometry in heart failure generally demonstrates a restrictive picture (Kee and Naughton 2010) with the reduction in lung volume likely due to increased cardiac size (Olson et al. 2006).

Furthermore, patients with chronic heart failure often have excess fluid which can lead to rostral fluid shift in the supine position (Yumino et al. 2010). This leads to pulmonary edema and orthopnea and has been hypothesized to contribute to central sleep apnea (Yumino et al. 2010). Increase lung fluid would result in interstitial edema, airways distortion, and even alveolar collapse potentially increasing ventilation heterogeneity.

By measuring increased ventilation heterogeneity, multiple-breath nitrogen washout (MBNW) has been used to detect early changes in small airway function in a variety of disease states including smoking, chronic obstructive pulmonary disease, bronchiolitis obliterans, and asthma (Verbanck et al. 1998, 2004, 2010; Thompson et al. 2013, 2014). The most widely used MBNW index is the lung clearance index (LCI) which provides a measure of global lung ventilation heterogeneity (Becklake 1952). More recently, the Sacin (for ventilation heterogeneity generated in the region of the acinar entrance) and Scond (for ventilation heterogeneity in the conductive lung zone) have been used to determine the ventilation heterogeneity in the peripheral (acinar) and proximal (conducting airways) compartments (Verbanck and Paiva 2011). The aim of this study was to determine the effect of chronic heart failure on ventilation heterogeneity. In addition, we sought to evaluate the effect of potential fluid shifts in this population.

## Materials and Methods

Study protocol was approved by the Alfred Ethics Committee (Project no 193/10). We recruited ambulatory patients with known CHF due to left ventricular systolic failure from our institution’s heart failure service as well as healthy controls. Heart failure patients were ambulatory and clinically stable.

Multiple-breath nitrogen washout (MBNW) was performed in both the seated and supine positions. Testing in the supine position was performed after 5 and 45 min of lying in the supine position. Testing at 5 min was done to control for any affect positional change would have of ventilation heterogeneity while testing was performed after 45 min based on data which suggest most lower limb fluid shifts have occurred in this time (Berg et al. 1993). MBNW testing was performed using a double “bag-in-box” system with participants instructed to breathe at 1 L tidal volumes (visual assistance provided via a computer display). Following a period of stable air breathing, participants were switched to breathing of 100% O_2_. Nitrogen concentration was continuously monitored at the mouth (AU9240-4032, Medical Graphics Corporation, St. Paul, MN), while flow and volume were calculated by volume displacement from within the double bag-in-box system and measured by pneumotachography (Fleish type, flow range 0–5 L·sec^−1^). Participants continued to breathe O_2_ until nitrogen was washed out to below 2%. This test was repeated three times in each position (a total of nine tests) with sufficient time allowed for complete re-equilibration of nitrogen within the lung functional residual capacity before subsequent testing was commenced (Robinson et al. 2013). MBNW data were analyzed using an automated process previously described by our group (Stuart-Andrews et al. 2012).

Following MBNW testing, subjects underwent spirometry, lung volume and TLCO measurement while seated in accordance with American Thoracic Society/European Respiratory Society guidelines (Macintyre et al. 2005; Miller et al. 2005; Wanger et al. 2005).

Statistical analyses were performed with the use of PASW Statistics 18 (SPSS Inc.; Chicago, IL) and results presented as mean ± standard deviation unless otherwise noted.

## Results

Thirty chronic heart failure patients and 10 healthy controls were recruited. One heart failure patient was found to have reversible airways obstruction on spirometry and a second patient was found to have bronchiectasis on CT chest, both were excluded from the analysis. Mean duration of heart failure diagnosis was 8.9 ± 8.5 years. Demographic and lung function data of the two groups are shown in Tables1 and 2. The lung function of the heart failure patients is consistent with those described in previous studies (Kee and Naughton 2010).

**Table 1:** Demographics

	Heart failure (*n* = 28)	Controls (*n* = 10)	*P* value
Age (years)	57 ± 10	51 ± 13	0.150
% Female	18	10	
Body mass index	32 ± 7	26 ± 4	0.020
% Ever smoked	57	20	
Cigarette pack years	17 ± 21	1 ± 2	0.001
New York Heart Association Class	2.4 ± 0.8	0 ± 0	<0.001

All data are presented as mean ± standard deviation.

**Table 2:** Lung function

	Heart failure (*n* = 28)	Controls (*n* = 10)	*P* value
Forced expiratory volume in 1 sec (% predicted)	83 ± 14	104 ± 14	<0.001
Forced vital capacity (% predicted)	85 ± 16	104 ± 14	0.003
Total lung capacity (% predicted)	97 ± 15	111 ± 13	0.016
Functional residual capacity (% predicted)	80 ± 18	94 ± 15	0.125
Residual volume (% predicted)	99 ± 24	93 ± 31	0.487
Transfer factor for the lung for carbon monoxide (% predicted)	80 ± 18	107 ± 12	<0.001
Maximal inspiratory pressure (% predicted)	83 ± 27	108 ± 36	0.044
Maximal expiratory pressure (% predicted)	92 ± 25	112 ± 22	0.047

All data are presented as mean ± standard deviation.

Chronic heart failure patients had a mean ejection fraction of 30 ± 12% on transthoracic echocardiogram (*n* = 20) and 32 ± 16% on gated cardiac blood pool scan (*n* = 12). Of these, seven patients had both tests while the three remaining patients had documented left ventricular dysfunction but no recorded left ventricular ejection fraction. Eight patients had a right heart catheter within a week of the MBNW. They had a pulmonary artery wedge pressure of 16 ± 8 mmHg (normal 2–15 mmHg), a cardiac index of 2.2 ± 0.4 L/min/m^2^ (2.6–4.2L/min/m^2^) and a pulmonary vascular resistance of 3.1 ± 1.1 Wood units (0.25–1.6 Woods units).

Heart failure patients were found to have higher LCI and Scond than controls. There was no significant difference in Sacin between CHF patients and control subjects (see Table3).

**Table 3:** Ventilation heterogeneity

	Heart failure (*n* = 28)	Controls (*n* = 10)	*P* value
Lung clearance index (lung turnovers)	9.6 ± 1.2	8.6 ± 1.4	0.034
Sacin (/L)	0.197 ± 0.171	0.125 ± 0.081	0.214
Scond (/L)	0.029 ± 0.014	0.006 ± 0.016	<0.001

All data are presented as mean ± standard deviation.

There was no change in any measure of ventilation heterogeneity after 5 and 45 min in the supine position in either group (see Table4). As expected, functional residual capacity changed significantly with posture, however there was no change with time in the supine position (see Table4).

**Table 4:** Effect of position

	Seated	Supine (5 min)†	Supine (45 min)†	*P* value*
Lung clearance index heart failure (lung turnovers)	9.6 ± 1.2	9.5 ± 1.7	9.4 ± 1.3	0.275
Lung clearance index controls (lung turnovers)	8.6 ± 1.4	8.4 ± 1.5	8.5 ± 0.7	0.593
Sacin heart failure (/L)	0.197 ± 0.171	0.157 ± 0.081	0.158 ± 0.087	0.168
Sacin controls (/L)	0.125 ± 0.081	0.108 ± 0.0.045	0.107 ± 0.044	0.383
Scond heart failure (/L)	0.029 ± 0.014	0.029 ± 0.020	0.030 ± 0.013	0.760
Scond controls (/L)	0.006 ± 0.016	0.007 ± 0.016	0.013 ± 0.013	0.332
Functional residual capacity heart failure (L)	2.000 ± 0.539	1.752 ± 0.504	1.752 ± 0.419	<0.001
Functional residual capacity controls (L)	2.380 ± 0.473	1.897 ± 0.329	1.888 ± 0.397	0.001

All data are presented as mean ± standard deviation.

* Paired *t*-test of seated versus supine 45 min.

† Two heart failure patients were unable to perform testing in the supine position and were excluded from this analysis.

**Figure 1:**
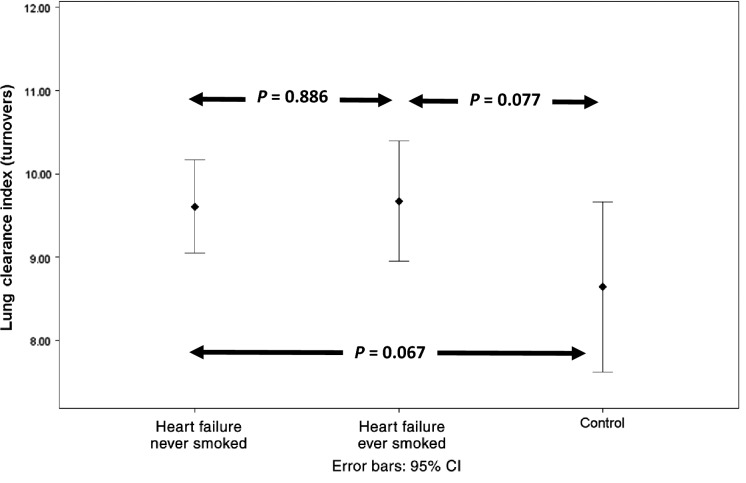
Comparison of lung clearance index in heart failure never smokers (*n* = 12), heart failure ever smokers (*n* = 16), and controls (*n* = 10).

**Figure 2:**
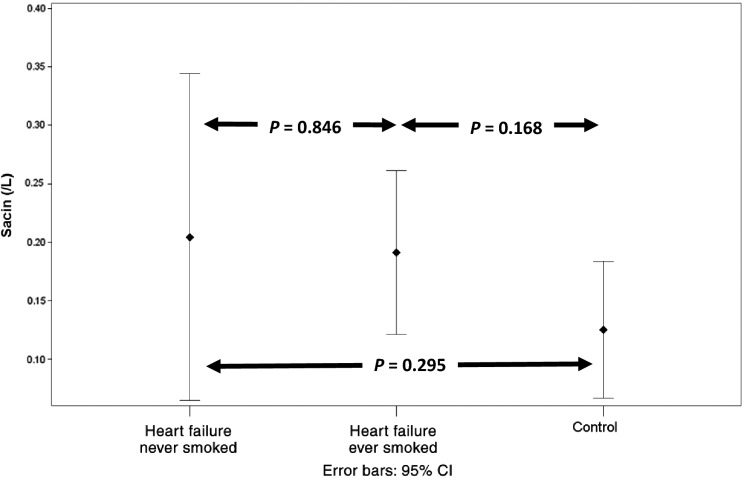
Comparison of Sacin in heart failure never smokers (*n* = 12), heart failure ever smokers (*n* = 16), and controls (*n* = 10).

**Figure 3:**
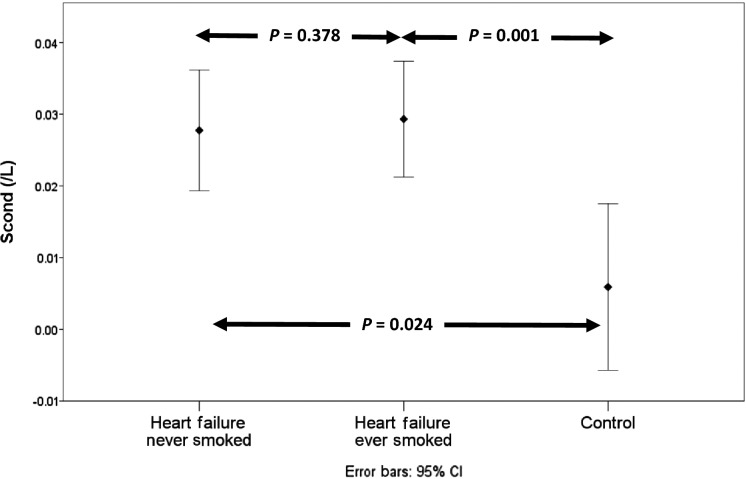
Comparison of Scond in heart failure never smokers (*n* = 12), heart failure ever smokers (*n* = 16), and controls (*n* = 10).

Dividing the heart failure patients into heart failure never smokers (*n* = 12) and heart failure ever smokers (*n* = 16) revealed no significant difference between the two subgroups in any measure of ventilation heterogeneity. There continued to be a difference in Scond between the heart failure nonsmokers and controls when the smokers were removed from the analysis, while LCI just failed to reach statistical significance most likely due to a loss of statistical power (see Figs.1–3).

There was a significant difference in body mass index between the two groups. It is unclear if body mass index affects ventilation heterogeneity, however there was no statistically significant correlation between body mass index and measures of ventilation heterogeneity in either the heart failure or control groups (see Table5). There was no correlation between body mass index and any measure of ventilation heterogeneity in the complete data set.

**Table 5:** Correlation with body mass index

	Lung clearance index	Sacin	Scond
Heart failure	*r* = −0.266, *P* = 0.172	*r* = −0.303, *P* = 0.117	*r* = 0.028, *P* = 0.888
Control	*r* = −0.094, *P* = 0.796	*r* = −0.161, *P* = 0.656	*r* = −0.292, *P* = 0.412

All data are presented as mean ± standard deviation.

## Discussion

This study demonstrates that chronic heart failure increases ventilation heterogeneity specifically at the level of the conducting airways. This is consistent with lung biopsy findings which demonstrated peripheral airway compression from dense connective tissue scarring in patients with mitral stenosis (Haworth et al. 1988). Surprisingly, there was no difference in Sacin between the two groups given that similar scarring has been described at the acinar level (Haworth et al. 1988). These findings suggest that CHF results in detectable abnormalities in airway function. Increased ventilation heterogeneity impairs regional ventilation–perfusion matching and leads to impaired gas exchange which may have clinical consequences in terms of the pathophysiology of dyspnea and exercise capacity.

Smoking has been shown to increase Scond and Sacin (Verbanck et al. 2004). Given the difference in smoking history between our heart failure and control groups it is possible that some of the difference in ventilation heterogeneity between the two groups was due to smoking. Dividing the heart failure group into never smokers and smokers demonstrated no statistically significant difference in measures of ventilation heterogeneity. Comparison of ventilation heterogeneity after removal of the smoking heart failure patients continued to demonstrate a significant difference in Scond (see Fig.1). These findings suggest that smoking history had little effect on ventilation heterogeneity and that the presence of heart failure is the primary cause of increased ventilation heterogeneity.

There was no change in ventilation heterogeneity in the supine position either at 5 or 45 min. This contrasts with a study that showed a change in ventilation heterogeneity measured by multiple-breath nitrogen washout in normal subjects moving from the standing to the supine position while in microgravity (Prisk et al. 1995). Other investigators have found no change in ventilation heterogeneity as measured by multiple-breath nitrogen washout comparing supine versus prone positions in normal subjects (Rodriguez-Nieto et al. 2002). While it is well recognized that posture affects ventilation distribution (West 2008), these data would suggest that it has little effect on ventilation heterogeneity.

We had hypothesized that in heart failure patients, rostral fluid shifts would play a more prominent role in altering ventilation heterogeneity especially after a prolonged period in the supine position. We did not however detect a change in ventilation heterogeneity in our subjects after 45 min in the supine position. Most rostral fluid shift from the lower limbs occurs within the first 45–60 min (Berg et al. 1993). Thus, our results suggest that either there was insufficient rostral fluid shift in our patient into the lungs to cause changes in ventilation heterogeneity or that lung fluid does not alter ventilation heterogeneity. We suspect the former is the case. Our patients were clinically euvolemic and, by necessity for this study, were able to lie flat for over 45 min. As well as this, there was no change in functional residual capacity with time spent in the supine position. This suggests that any fluid shift from spending time in the supine position was relatively mild as significant fluid shift into the lung would likely result in a fall in functional residual capacity. Unfortunately measures of fluid shift such as change in calf circumference or conductance were not collected during our study. Our study does not rule out changes in ventilation heterogeneity in the supine position with greater fluid overload. Spirometry in patients with acute pulmonary edema or in whom normal saline has been given as a rapid infusion has demonstrated evidence of airways obstruction suggesting sufficient lung fluid will affect ventilation (Sharp et al. 1958; McNicol et al. 1965; Light and George 1983). The lack of change in our study does suggest that the increase in lung clearance index and Scond found in our heart failure subjects compared to controls are the result of structural change within the lung and not due to excess lung fluid.

One weakness of our study is that our heart failure patients had a significantly greater body mass index than the controls. Obesity may increase ventilation heterogeneity however it has been shown that ventilation heterogeneity when measured by the forced oscillation technique is only altered by obesity when it is severe enough to cause lung volumes to fall below 65% predicted (Pellegrino et al. 2014). Given our patients had relatively mild obesity and relatively preserved lung volumes, it is unlikely that weight had a significant effect on their ventilation heterogeneity. This is further confirmed by the lack of correlation between body mass index and ventilation heterogeneity in our subjects (Table5).

Another weakness of our study is that we did not have a direct histopathological or radiological measure of the parenchymal changes we have proposed as a reason for the increased ventilation heterogeneity in heart failure. Given such changes were described at an electron microscope level in lung biopsy specimens it is unlikely they could be quantified using radiological imaging alone. Obtaining lung biopsy specimens for a study such as ours would be unnecessarily invasive. TLCO has been shown to be a good measure of the pulmonary vascular changes which occur in association with parenchymal change (Aber and Campbell 1965). In our study, LCI and Scond in CHF both increased with falling TLCO but this failed to reach statistical significance (*P* = 0.119 and *P* = 0.087, respectively).

## Conclusions

Ventilation heterogeneity (lung clearance index and Scond) is increased in chronic heart failure. Supine position does not affect ventilation heterogeneity in either heart failure or controls. Further studies are required to determine the clinical impact of increased ventilation heterogeneity in these patients.
